# The Rheumatism of Kalmia

**Published:** 1891-04

**Authors:** S. Lilienthal


### THE RHEUMATISM OF KALMIA.

Editors Homœopathic Physician:

C. Hering says, in the *Guiding Symptoms* and in Farrington's *Hering's Condensed*, article Kalmia-lat., that the rheumatic pains generally go from upper to lower extremities, or shift about suddenly, while Farrington, in his *Clinical Materia Medica*, page 357, says: "The Kalmia rheumatism, like that of Ledum, almost always travels upward." Lippe, in his *Materia Medica*, page 341, sides with Hering: "The rheumatism generally goes from the upper to the lower extremities." Guerin Mineville (*Matiere Med.*, II, 271) also says: "Les douleurs marchent la plus habituellement de haut en bas." Dunham is silent about this point, but emphasizes the weakness characteristic of this drug. The provers, as we see in the *Cyclopædia of Drug Pathogenecy*, neglect to inform us about it. And still I am loth to give up Farrington, especially as he says, "like that of Ledum, which we know to travel upward." The paralytic symptoms of Conium travel upward and the paresis of Kalmia may do the same. Will our learned men inform our readers about this point. Alas! I am always in trouble about our materia medica pura.

S. Lilienthal.

